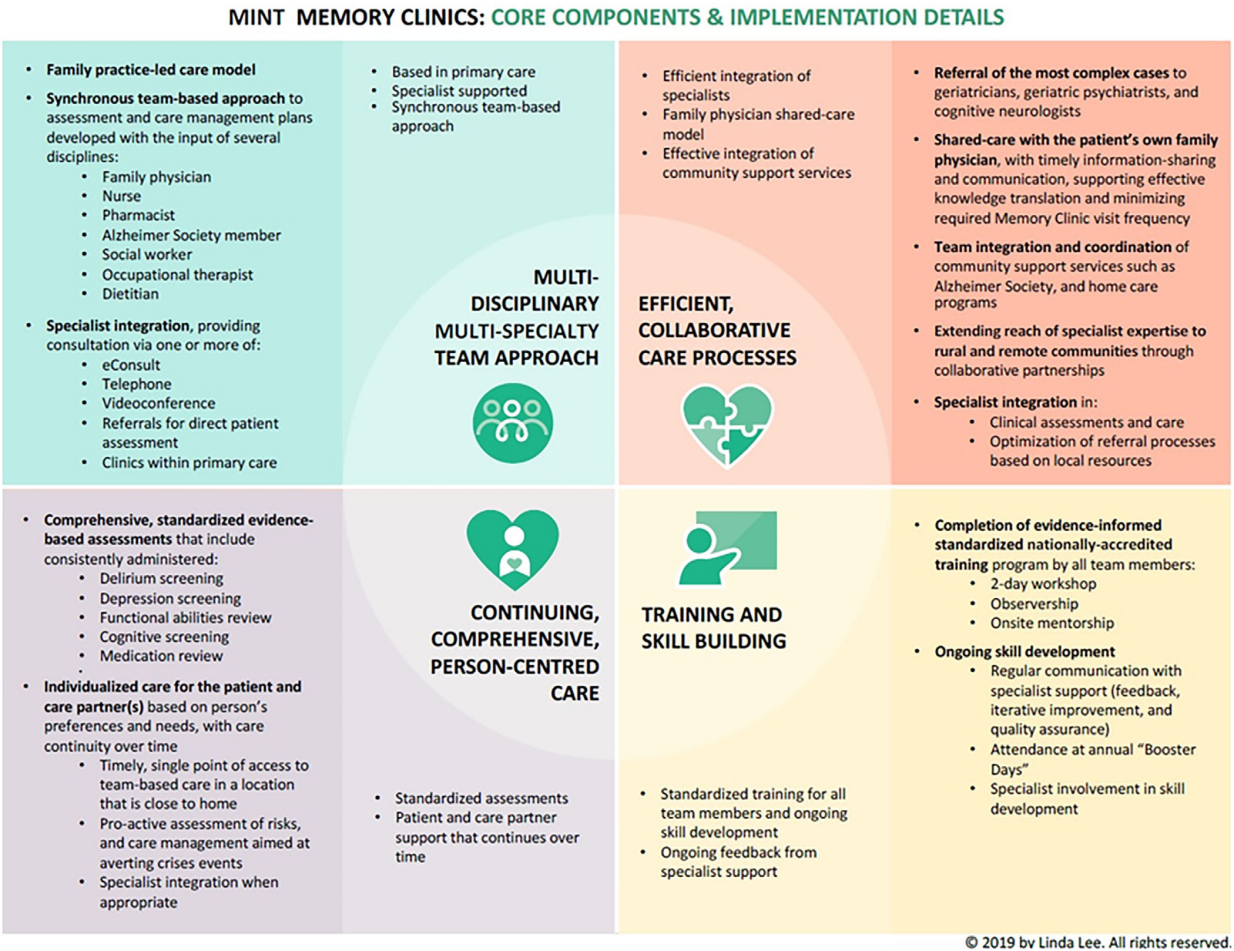# Multispecialty Interprofessional Team (MINT) Memory Clinic Overview and Data From Spread Across Canada

**DOI:** 10.1002/alz.087434

**Published:** 2025-01-09

**Authors:** Linda Lee, Loretta M Hillier, Michael Lee

**Affiliations:** ^1^ McMaster University, Kitchener, ON Canada; ^2^ MINT Memory Clinic, Kitchener, ON Canada; ^3^ Schlegel‐UW Research Institute on Aging, Waterloo, ON Canada; ^4^ Geras Centre for Aging Research, Hamilton, ON Canada

## Abstract

**Background:**

Multispecialty Interprofessional Team (MINT) Memory Clinics build capacity for dementia care within primary care. This presentation will provide an overview of the MINT care model and results of a process evaluation of the implementation of the model in three provinces in Canada using the Research Medical Council framework for evaluating complex interventions.

**Methods:**

178 healthcare providers (HCP) were trained to establish 10 MINT clinics across three Canadian provinces. We collected data on clinic referrals and service provision over a nine‐month period to describe fidelity (assessment adherence; management; timely access), dose (number of assessments, follow‐up appointments), and reach (patients served). We conducted individual interviews with 20 key informants (HCP, system leaders) to learn about the contextual factors that affected implementation and clinic impacts.

**Results:**

Across 10 clinic sites, 521 patients were referred; 368 (71%) completed assessments. All clinics implemented the assessment protocol as fully intended with the exception of two clinics which did not administer depression and caregiver burden screening. The majority of patients (73%) were assessed within a month of referral; 87% of patients were managed in primary care, with 13% referred for specialist consultation. Follow‐up appointments were recommended for 83% of patients assessed (305/368); 33% (N = 123) completed follow‐up appointments. Key themes related to context were: MINT model fits an existing care gap; Multi‐disciplinary collaborative approach; Systematic approach to diagnosis and management; and, the need for easily accessible care close to home. Facilitating factors included: Senior leadership and physician champion support; Access to and support from various disciplines; and Access to resources (funding, space). Identified challenges varied by province and included: Lack of resources (staff, funding, space) and HCP new to dementia care required time to assimilate new knowledge to practice. The clinics were seen as impacting dementia care with: Provision of better dementia care than usually available in primary care; More comprehensive assessment and care plans; Interprofessional collaboration; Increased care partner support, and Quicker access to assessment and diagnosis.

**Conclusions:**

The implementation of the MINT model in three provinces with different health systems demonstrates its scalability and its potential to improve dementia care in differing jurisdictions.